# Combined Analysis of SNP Array Data Identifies Novel CNV Candidates and Pathways in Ependymoma and Mesothelioma

**DOI:** 10.1155/2015/902419

**Published:** 2015-06-22

**Authors:** Gabriel Wajnberg, Benilton S. Carvalho, Carlos G. Ferreira, Fabio Passetti

**Affiliations:** ^1^Bioinformatics Unit, Clinical Research Coordination, National Cancer Institute of Brazil (INCA), 20231-050 Rio de Janeiro, RJ, Brazil; ^2^Graduate Program in Systems and Computational Biology, Oswaldo Cruz Institute, Oswaldo Cruz Foundation (Fiocruz), 21040-360 Rio de Janeiro, RJ, Brazil; ^3^Laboratory of Functional Genomics and Bioinformatics, Oswaldo Cruz Institute, Oswaldo Cruz Foundation (Fiocruz), 21040-360 Rio de Janeiro, RJ, Brazil; ^4^Department of Medical Genetics, School of Medical Sciences, State University of Campinas, 13083-887 Campinas, SP, Brazil; ^5^Clinical Research Coordination, National Cancer Institute of Brazil (INCA), 20231-050 Rio de Janeiro, RJ, Brazil

## Abstract

Copy number variation is a class of structural genomic modifications that includes the gain and loss of a specific genomic region, which may include an entire gene. Many studies have used low-resolution techniques to identify regions that are frequently lost or amplified in cancer. Usually, researchers choose to use proprietary or non-open-source software to detect these regions because the graphical interface tends to be easier to use. In this study, we combined two different open-source packages into an innovative strategy to identify novel copy number variations and pathways associated with cancer. We used a mesothelioma and ependymoma published datasets to assess our tool. We detected previously described and novel copy number variations that are associated with cancer chemotherapy resistance. We also identified altered pathways associated with these diseases, like cell adhesion in patients with mesothelioma and negative regulation of glutamatergic synaptic transmission in ependymoma patients. In conclusion, we present a novel strategy using open-source software to identify copy number variations and altered pathways associated with cancer.

## 1. Introduction

Many research groups have studied human genomic diversity, including various types of DNA sequence alterations, such as copy number variation [1]. Among other possible definitions, DNA copy number variation (CNV) can be described as “a copy number change involving a DNA fragment that is ~1 kilobase (kb) or larger” [1]. Here, we use CNV in the context of structural changes in DNA copy number variation. Despite the constant improvements in the high-throughput sequencing (HTS) technology, it is still challenging to use SNP array data to search for novel structural CNVs [1].

Array-based Comparative Genomic Hybridization (aCGH) is a technique developed exclusively to detect amplifications and losses. On the other hand, researchers currently use microarrays targeting millions of Single Nucleotide Polymorphisms (SNPs) to perform both genotyping and copy number analyses [2]. The allele-specific probes present in SNP chips allow the researchers to quantify not only the relative allelic abundance through the computation of log-ratios [3] but also the total locus-specific abundance [4]. These statistics are then used to obtain genotypes and a higher resolution CNV landscape, if compared to aCGH data.

Affymetrix designed a number of arrays suitable for copy number analysis. These designs differ essentially in their densities, ranging from 10 thousand to 2.7 million markers. Researchers use the genome-wide SNP 6.0 (1.8 million markers) and the CytoScan HD (2.7 million markers) arrays for current copy number studies [5]. However, it is not uncommon to identify a significant number of investigations that used the 500K chipset, comprised of two 250K designs based, respectively, on the Nsp and Sty restriction enzymes.

One tool used for analysis of CNV data using Affymetrix arrays is the Copy Number Analysis Tool (CNAT) [6]. CNAT uses an extension of a Robust Linear Model with the Mahalanobis distance classifier algorithm (RLMM) known as BRLMM. This algorithm adds a Bayesian step that provides an improved estimate of cluster centers and variances [7]. A noncommercial option usually used is the Copy Number Analyzer for Affymetrix GeneChip Mapping arrays (CNAG). However, the source codes for CNAT and CNAG are not available. Therefore, the scientific community cannot suggest modifications that would make the software suitable for specific requirements of each research project.

Novel analyses of CNV using publicly available microarray data from tumor samples are sparse. One such study analyzed data from expression arrays from hepatocellular carcinoma patients and identified newly coexpressed genes in tumor and adjacent normal tissues using unsupervised clustering [8]. Another study identified chromothripsis-like patterns from 918 published microarray cancer samples [9]. These examples demonstrate the potential in developing innovative strategies to analyze published datasets, culminating in novel findings to the scientific community.

In this paper, we present a novel strategy to identify structural CNVs using Affymetrix Nsp 250k data. We examined two published cancer datasets using two complementary Bioconductor alternatives for CNV data analysis: DNAcopy [10] and CGHcall [11]. We identified novel regions, genes, and pathways associated with mesothelioma and ependymoma, corroborating the original findings [12, 13].

## 2. Materials and Methods

### 2.1. Samples

We analyzed two different cancer datasets based on Affymetrix Nsp 250k SNP array, distributed through the NCBI Gene Expression Omnibus (GEO) [14] service. Both datasets refer to matched-pair DNA samples (tumor and peripheral blood). One group studies 23 mesothelioma patients (GEO accession GSE20989) [12], while the other investigates 40 ependymoma patients (GEO accession GSE32101) [13].

### 2.2. Data Analysis

We analyzed the data using the statistical analysis software R (version 2.14.0) [15] and Bioconductor (version 2.11) [16] packages. We used the oligo package (version 1.18.1) [17] to import, preprocess, and genotype CEL files via the Corrected Robust Linear Model with Maximum Likelihood Distance (CRLMM) algorithm [3]. CRLMM uses SNPRMA, an adapted version of the Robust Multiarray Average (RMA) algorithm, to preprocess SNP data. We annotated the genotyped probe sets using information from the pd.mapping250k.nsp package, based on the human genome (hg18) reference.

To remove the biological noise, we used the following expression:

(1) $$\begin{array}{c} FC=\log\left(\;\frac{T}{N}\;\right), \end{array}$$

where *FC* corresponds to the log-ratio for each probe set, *T* represents the signal of the tumoral sample, and *N* indicates the signal for the paired peripheral blood sample.

We segmented the log-ratio data using the Circular Binary Segmentation (CBS) algorithm, distributed through the Bioconductor DNAcopy package (version 1.28) [10]. These segments represent numerically regions that share the same relative copy number. We used the thresholds set by Christensen and colleagues [12] and Kilday and colleagues [13] to call gains and losses: at least 0.5 for amplified regions and at most −0.5 for a lost region. We also combined the segmentation results with the CGHcall Bioconductor package (version 2.14) [11]. This allowed us to estimate the probability of a given segment being classified as an amplification or a loss. With CGHcall, we only considered segments with a probability higher than 80% to classify it as an amplified or lost region.

We used the MGSA package (version 1.13) [18] to perform gene set enrichment analysis (GSEA) and search for enriched pathways. We used two different databases for GSEA: Gene Ontology (GO) [19] and KEGG [20]. We used Cytoscape (version 3.1.1) [21] and the Reactome database [22] to build the functional interaction networks of genes participating in GO pathways.

## 3. Results

### 3.1. Mesothelioma Dataset

**Figure 1:**
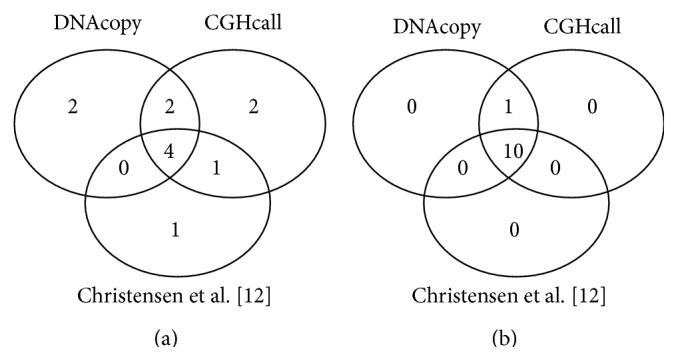
Venn diagram comparing the number of chromosomal regions in which gained (a) or lost (b) regions were identified by DNAcopy and CGHcall and by Christensen and colleagues [12].

Christensen and colleagues [12] used CNAT to identify CNVs on the 23 mesothelioma patients. They grouped their results by chromosomal arms and we used the same strategy to compare our results to theirs (Tables 1 and 2). We used both DNAcopy and CGHcall to identify altered copy number segments within regions reported by Christensen et al. For example, we identified CTNND2 amplification in 2 patients within the 5p amplified region using CGHcall (Table 1). Another example is the identification, using DNAcopy, of 10 patients with losses in the 9p chromosomal arm, which includes the* CDKN2A *and* CDKN2B* tumor suppressor genes (43% of cases). Within the 16q region, we identified the loss of both copies of* CDH8* (found in 6 patients using both packages),* CDH11*,* JAM3*, and* NCAM* genes (all three found in 4 patients using both packages). We were also able to identify novel undetected regions that had been amplified: 10p and 6q. The lost 10p region includes the gene* FZD8* (Figure 1(a)). Regarding the lost regions, we ascertained that our approach detected the chromosomal arm 16q with high frequency. Our strategy detected all 10 lost regions identified by Christensen and colleagues [12], using both DNAcopy and CGHcall packages. Using their findings as reference, our approach allowed us to identify novel CNVs (Table 2 and Figure 1(b)).

**Figure 2:**
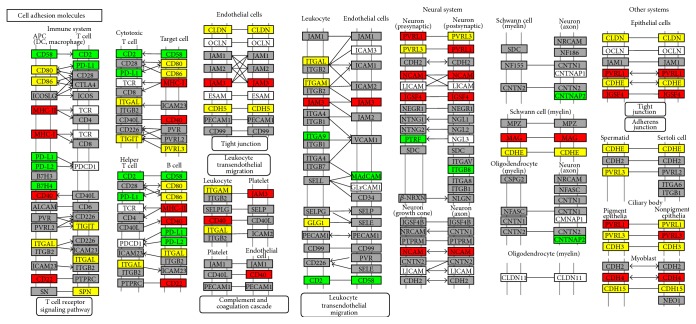
KO:04514 from KEGG: colored if genes were identified in at least one patient with amplification (green), lost (red), both (yellow), and with probe with no hybridization (gray).

We also conducted an analysis to detect altered metabolic pathways in this mesothelioma dataset. We identified cell adhesion molecules (CAMs) (KO:04514) (Figure 2) as altered with a probability of 76% and 93% of adherens junction organization (GO:0034332) as affected. We observed that the following pathways were altered with high probability: MHC class II protein complex (96%) (GO:0042613) and the Fanconi anemia pathway (86%) (KO:03460) (Table 3).

### 3.2. Ependymoma Dataset

Kilday and colleagues [13] used the CNAG [23] to identify CNVs grouped in the chromosomal arms using 40 intracranial ependymoma samples. Applying the same approach, we used chromosomal arms as a reference to compare the results (Tables 3 and 4). We identified 35 amplified regions. The 1q region was the most frequently amplified, found in 22.5% of patients using DNAcopy. The* ADORA1* gene was present in extra copies in 87% of the patients who presented amplifications at the 1q region. We also identified 12 lost regions with DNAcopy. The chromosomal arm 2q was lost in 27.5% of the patients.

Using the CGHcall package, we detected 35 amplified regions. Regions with the highest amplification rates were 1p (15 patients), 18q and 20q (13 patients), 18p, 19p, and 20p (12 patients), and 21q (11 patients). In the 20q region, we identified the amplification of the cadherin gene* CDH22* in 13 patients (when using CGHcall) and 4 patients (while using DNAcopy).

**Figure 3:**
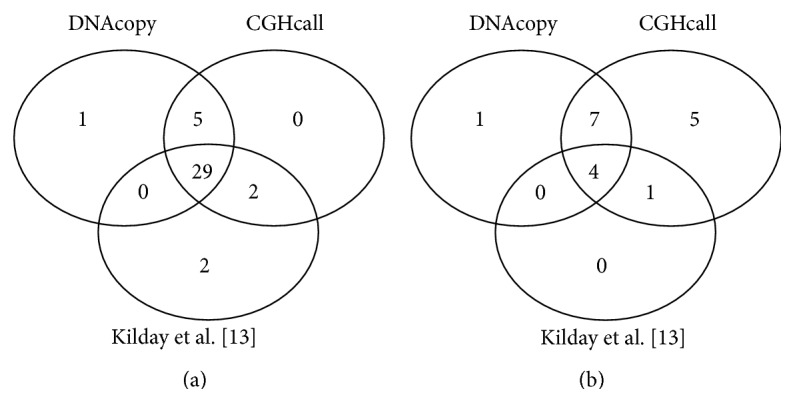
Venn diagram comparing the number of chromosomal regions in which gained (a) or lost (b) regions were identified by DNAcopy and CGHcall and by Kilday and colleagues [13].

Considering only the 17 lost regions identified by CGHcall, the most frequently lost chromosomal arms were 1p (14 patients) and 2q and 6q (11 patients). Among all 33 amplified regions identified by Kilday and colleagues [13], we identified 29 of them with both packages. However, we could not detect the region 14p nor the region 21p. We identified all of the 5 lost regions identified by Kilday and colleagues [13]. Using the DNAcopy package, we observed that 5 patients lost copies of the* PLA2G6* gene (22q region). This number increases to 7 patients when analyzing the data with the CGHcall package, as shown by Tables 4 and 5 and Figure 3.

**Figure 4:**
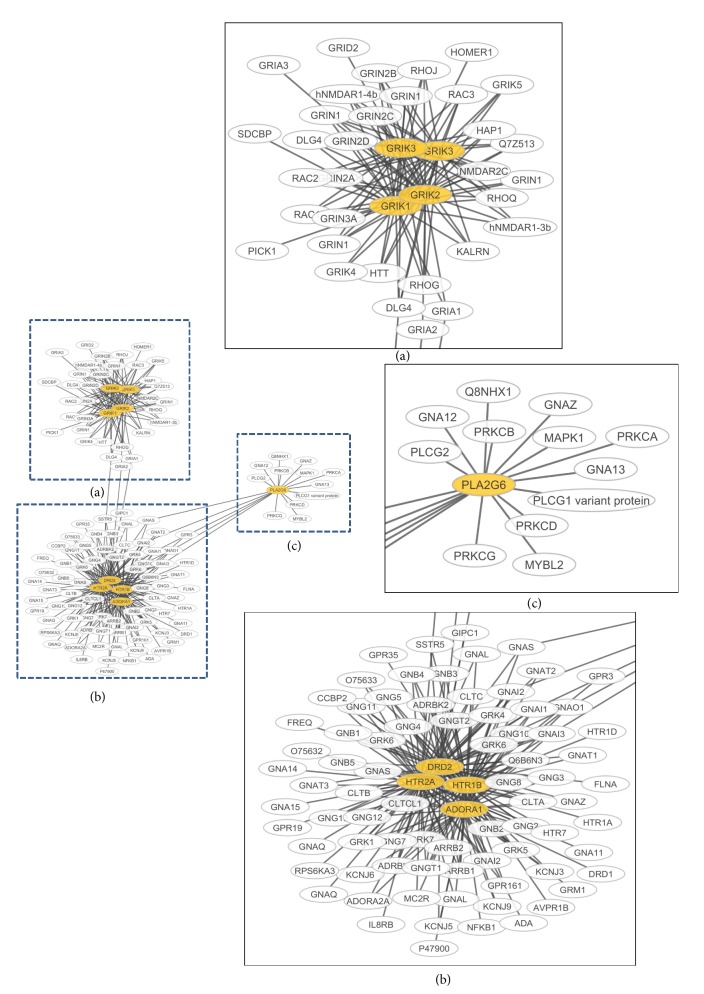
Functional interaction of genes with amplifications (yellow) in the “negative regulation of synaptic transmission, glutamatergic” GO pathway (GO:0051967).

Our GSEA of this dataset identified statistically significant altered pathways, as follows: (1) calcium-dependent cell-cell adhesion pathway (GO:0016339), probability of 73%; (2) protein digestion and absorption (KO:04974), probability of 66%; and (3) negative regulation of synaptic transmission, glutamatergic (GO:0051967), probability of 55% (Table 6 and Figure 4). We used Cytoscape to evaluate which genes would be affected by the impact of the genes with CNVs in the negative regulation of synaptic transmission, glutamatergic pathway and we found three clusters (Figure 4). We noticed that two groups were characterized by the interaction of more than 15 protein-coding genes: the* GRIK* gene family members (*GRIK1*,* GRIK2*, and* GRIK3*) (Figure 4(a)) and the* ADORA1*,* HTR1B*,* HTR2A*, and* DRD2 *(Figure 4(c)).

## 4. Discussion

Storing and distributing published datasets through public databases allows researchers to reanalyze the data using up-to-date methodological strategies. This approach can reveal novel findings, aggregating value to the scientific knowledgebase at lower costs.

On the mesothelioma microarray dataset, we identified five of the 6 amplified regions detected by Christensen and colleagues [12] applying the CGHcall package and four using the DNAcopy package. We observed four undetected amplified chromosome arms when our approach was combined with the CGHcall package. We also detected the* FDZ8* gene associated with the lost chromosomal arm 10p; this gene has been previously associated with resistance to chemotherapy in breast cancer patients [24]. When we used the CGHcall package, we also identified two patients with extra copies of the* CTNND2* gene, which encodes a protein that promotes the disruption of the adhesion protein E-cadherin, favoring cell migration and therefore cancer metastasis [25].

Christensen and colleagues [12] identified ten lost chromosomal arms in mesothelioma patients. We detected eleven affected chromosomal arms in our analysis, and eight of these lost regions are the same as detected by Christensen's group. One of these lost regions was 9p, where the tumor suppressor genes* CDKN2A* and* CDKN2B* are located. These genes encode two proteins that inhibit the CDK4 protein preventing continuation of the cell cycle in G1 [26]. Some genes for adhesion molecules with lost copies have been identified, for example, the cadherins* CDH8 *(6 patients) and* CDH11* (4 patients). The cadherin genes are located in the 16q arm and loss of heterozygosity was previously reported in nephroblastoma, hepatocellular carcinoma, prostate cancer, and breast cancer [27]. Cadherins are important tight junction molecules and the absence of these molecules can promote cancer metastasis [28]. We observed the loss of the* NCAM* gene in 4 patients: this can affect cell-matrix adhesion and stimulate cell migration [29] (Tables 1 and 2). CNVs in adhesion genes may alter pathways implicated in cell adhesion, as our GSEA using the KEGG and GO databases suggests (Table 3 and Figure 2). The CNVs identified in our analysis could be associated with the lack of the expression of adhesion proteins in human mesothelioma cell lines that had been previously described [30]. Therefore, our combined approach not only replicated results published by Christensen and colleagues but also provided additional support that reduced cell adhesion in mesothelioma could be used as a target to improve patient treatment.

Another work studied CNVs in 22 mesothelioma patients and identified lost copies of* CDKN2A* and* CDKN2B* tumor suppressor genes [31]. We also identified lost copies of these two genes. Additionally, our combined approach detected CNV events at other genes associated with mesothelioma: lost copies of* LATS1* (associated with hippo signaling) and* NF2* (a tumor suppressor) and the amplification of the* YAP* gene, responsible for encoding a protein that activates transcription factors [32].

On the ependymoma dataset, we noticed that combining CGHcall and DNAcopy increased our ability to detect amplifications in the 1q chromosomal arm for most of patients, corroborating the original findings [13]. Kilday and colleagues [13] also identified a specific amplification of the 1q25 region, in which the* QSOX1* gene was associated with poor survival from the disease. We identified amplifications in the 1q25 region in 7 patients as well as in the gene* QSOX1* with the analysis through CGHcall. When using DNAcopy, we detected other events in the 1q region. With CGHcall, we identified all five lost regions identified by Kilday and colleagues [13]; we observed only four of them when using DNAcopy. We also detected amplification of the* ADORA1* gene (1q32 region) in 13 patients using CGHcall and in 8 patients using DNAcopy (Tables 4 and 5). This Adenosine A1 receptor participates in several signaling pathways. In the breast cancer MCF-7 cell line, the silencing of the* ADORA1* gene decreases the endogenous estrogen receptor-*α* (ER-*α*) levels and causes a decline in cell proliferation [33]. However,* ADORA1* plays an important role as a neuroprotective molecule in the central nervous system and its activation is a common target for drugs that treat neurodegenerative diseases (such as Alzheimer's disease and multiple sclerosis). The main result of ADORA1 receptor activation is the inhibition of the glutamate synapse [34]. Glutamate is a known apoptosis inducer and its use in ependymoma tumors can suppress tumor growth [35]. The release of glutamate by brain tumor cells has been associated with epileptic events in glioma patients. The system *x*_*c*_^−^, a plasmatic membrane cysteine/glutamate antiporter heterodimer, was identified as responsible for this glutamate release in ependymoma [36]. It is composed of two proteins, one encoded by the gene* SLC7A11 *and another by the gene* SLC3A2* [37]. In a clinical study, the most often reported clinical presentation in ependymoma patients was seizure and medically intractable epilepsy [38]. However, studies concerning glutamate release and ependymoma are not available. We detected amplification for the glutamate receptors* GRIK1*,* GRIK2*, and* GRIK3* (Figure 4(a)). We noticed that* SLC7A11*, one of the system *x*_*c*_^−^ genes, was amplified in 5 patients, which is evidence for the role of glutamate in ependymoma. Our combined analysis detected the loss of the* PLA2G6* gene (5 patients with DNAcopy and 7 patients with CGHcall).* PLA2G6* encodes the iPLA2 protein that phosphorylates the AMPA receptor (a glutamate receptor) [39]. According to our GSEA results, negative regulation of glutamatergic synaptic transmission was detected with a probability greater than 50% (Table 6), which can be interpreted as a novel pathway for investigation in ependymoma tumors.

In the ependymoma dataset, we identified additional altered pathways with high probability to occur (Table 6). Calcium-dependent adhesion mediated by E-cadherin is deficient in this type of tumor because of its low expression [40] and we found a 73% probability that this pathway was altered. We also detected extra copies of the* CDH22* gene in 13 patients with CGHcall and in 9 patients with DNAcopy. The overexpression of these genes in this pathway has been previously associated with tumor progression in colorectal cancer [41].

Comparing our findings for ependymoma with another study [42] that used the same array design as [13] and only the DNAcopy package to perform their analysis, we observed that some CNVs were identified exclusively by our approach. For example, when comparing with the findings of Johnson and colleagues [42], only our approach was able to identify lost copies of* SCHIP-1* (a NF2 protein interaction gene), despite the fact that the* NF2* genes have been previously described in ependymoma [43]. Ependymoma also has well-known amplified genomic regions [43], but only a gain of the* TERT* gene was identified by Johnson's group [42] as well as by our methodology. However, our combined approach identified novel potential biomarker candidates in ependymoma with a known relationship to cancer:* ERRB2*,* EGFR*,* TWIST1*,* CDK4*,* HDAC9*, and* ARHGEF5* genes.

Taken together, our results provide additional verification of novel and known pathways and molecular targets for the improvement of current treatments of ependymoma and mesothelioma.

## 5. Conclusions

We developed a novel combined approach using two different software packages from the Bioconductor Project (DNAcopy and CGHcall) to identify CNVs from SNP array data. We verified the accuracy of our methodology using two different previously published datasets that used the Affymetrix Nsp 250K arrays. We obtained results similar to those originally reported. However, our methodology also identified novel CNVs and possibly altered pathways. These pathways have strong biological background and can be further investigated as potential drug targets in mesothelioma and ependymoma.

## Figures and Tables

**Table 1:** Comparison of SNP array data of mesothelioma [12] analyzed by DNAcopy and CGHcall with amplifications.

DNAcopy		CGHcall		Christensen et al. [12]	
Chromosome arm	Patients	Chromosome arm	Patients	Chromosome arm	Patients
1q	6	1q	5	1q	7
15q	7	15q	5	15q	4
8q	4	8q	3	8q	9
7p	3	7p	4	7p	5
8p	4	5p	3	5p	5
10p	4	10p	4	10p	0
9p	0	9p	4	9p	0
6q	3	6q	3	6q	0
6p	0	6p	4	6p	0

**Table 2:** Comparison of SNP array data of mesothelioma [12] analyzed by DNAcopy and CGHcall with lost regions.

DNAcopy		CGHcall		Christensen et al. [12]	
Chromosome arm	Patients	Chromosome arm	Patients	Chromosome arm	Patients
1p	18	1p	16	1p	15
6q	15	6q	8	6q	15
22q	6	22q	10	22q	10
14q	9	14q	11	14q	9
9p	10	9p	11	9p	9
4q	8	4q	9	4q	7
4p	7	4p	6	4p	6
13q	4	13q	5	13q	4
18q	2	18q	13	18q	3
10p	2	10p	2	10p	2
16q	15	16q	6	16q	0

**Table 3:** Gene set enrichment analysis using MGSA package with posterior probability ≥0.5 for the mesothelioma dataset.

ID	Pathway	Posterior probability
KO:04740	Olfactory transduction	0.98
GO:0042613	MHC class II protein complex	0.97
GO:0034332	Adherens junction organization	0.93
KO:03460	Fanconi anemia pathway	0.86
KO:00350	Tyrosine metabolism	0.79
KO:00565	Ether lipid metabolism	0.76
KO:04514	Cell adhesion molecules (CAMs)	0.69
KO:00650	Butanoate metabolism	0.53
KO:05416	Viral myocarditis	0.53

KO IDs are from the KEGG database and GO IDs are from the Gene Ontology database.

**Table 4:** Comparison of SNP array data of ependymoma [13] analyzed by DNAcopy and CGHcall with amplifications.

DNAcopy		CGHcall		Kilday et al. [13]	
Chromosome arm	Patients	Chromosome arm	Patients	Chromosome arm	Patients
1q	9	1q	15	1q	14
2q	7	2q	9	2q	11
2p	4	2p	10	2p	11
19p	4	19p	12	19p	10
5p	5	5p	9	5p	10
17q	4	17q	7	17q	5
18p	3	18p	12	18p	10
17p	2	17p	7	17p	5
19q	3	19q	10	19q	9
15q	4	15q	13	15q	8
6q	5	6q	10	6q	8
6p	5	6p	9	6p	8
12q	5	12q	8	12q	6
21q	5	21q	11	21q	6
22q	6	22q	12	22q	7
7q	4	7q	9	7q	7
7p	4	7p	8	7p	7
9p	5	9p	9	9p	7
9q	5	9q	9	9q	7
20p	4	20p	12	20p	6
20q	4	20q	13	20q	6
11q	5	11q	5	11q	7
12p	5	12p	8	12p	6
8p	4	8p	6	8p	6
8q	5	8q	6	8q	6
4p	4	4p	9	4p	6
4q	5	4q	9	4q	6
14q	6	14q	9	14q	8
13q	7	13q	7	13q	0
3q	6	3q	7	3q	0
3p	4	3p	7	3p	0
11p	0	11p	11	11p	7
5q	0	5q	10	5q	10
10q	6	10q	10	10q	0
10p	4	10p	9	10p	0
18q	4	18q	0	18q	10

**Table 5:** Comparison of SNP array data of ependymoma [13] analyzed by DNAcopy and CGHcall with lost copies.

DNAcopy		CGHcall		Kilday et al. [13]	
Chromosome arm	Patients	Chromosome arm	Patients	Chromosome arm	Patients
6p	8	6p	10	6p	9
6q	9	6q	11	6q	8
22q	6	22q	7	22q	6
16q	6	16q	7	16q	6
3q	8	10q	7	10q	7
2q	11	2q	11	2q	0
14q	7	14q	7	14q	0
21q	7	21q	7	21p	0
3p	6	3p	8	3p	0
13q	6	13q	9	13q	0
9q	9	9q	9	9q	0
9p	0	9p	7	9p	0
11q	0	11q	7	11q	0
5q	0	5q	8	5q	0
1p	9	1p	14	1p	0
3q	0	3q	8	3q	0
11p	0	11p	9	11p	0
10p	0	10p	6	10p	0

**Table 6:** Gene set enrichment analysis using MGSA package with posterior probability ≥ 0.5 for the ependymoma dataset.

ID	Pathway	Posterior probability
GO:0004522	Pancreatic ribonuclease activity	0.97
GO:0042613	MHC class II protein complex	0.84
KO:04740	Olfactory transduction	0.79
GO:0016339	Calcium-dependent cell-cell adhesion	0.73
KO:04974	Protein digestion and absorption	0.66
GO:0046703	Natural killer cell lectin-like receptor binding	0.65
GO:0051967	Negative regulation of synaptic transmission, glutamatergic	0.55

KO IDs are from the KEGG database and GO IDs are from the Gene Ontology database.

## References

[1] Teo S. M., Pawitan Y., Ku C. S., Chia K. S., Salim A. (2012). Statistical challenges associated with detecting copy number variations with next-generation sequencing. *Bioinformatics*.

[2] Rauch A., Rüschendorf F., Huang J. (2004). Molecular karyotyping using an SNP array for genomewide genotyping. *Journal of Medical Genetics*.

[3] Carvalho B., Bengtsson H., Speed T. P., Irizarry R. A. (2007). Exploration, normalization, and genotype calls of high-density oligonucleotide SNP array data. *Biostatistics*.

[4] Wang W., Carvalho B., Miller N. D., Pevsner J., Chakravarti A., Irizarry R. A. (2008). Estimating genome-wide copy number using allele-specific mixture models. *Journal of Computational Biology*.

[5] Liu G., Loraine A. E., Shigeta R. (2003). NetAffx: affymetrix probesets and annotations. *Nucleic Acids Research*.

[6] Slater H. R., Bailey D. K., Ren H. (2005). High-resolution identification of chromosomal abnormalities using oligonucleotide arrays containing 116,204 SNPs. *The American Journal of Human Genetics*.

[7] Affymetrix BRLMM: an Improved Genotype Calling Method for the GeneChip Human Mapping 500K Array Set. http://media.affymetrix.com/support/technical/whitepapers/brlmm_whitepaper.pdf.

[8] Ge X., Yamamoto S., Tsutsumi S. (2005). Interpreting expression profiles of cancers by genome-wide survey of breadth of expression in normal tissues. *Genomics*.

[9] Cai H., Kumar N., Bagheri H. C., von Mering C., Robinson M. D., Baudis M. (2014). Chromothripsis-like patterns are recurring but heterogeneously distributed features in a survey of 22,347 cancer genome screens. *BMC Genomics*.

[10] Venkatraman E. S., Olshen A. B. (2007). A faster circular binary segmentation algorithm for the analysis of array CGH data. *Bioinformatics*.

[11] van de Wie M. A., Kim K. I., Vosse S. J., van Wieringen W. N., Wilting S. M., Ylstra B. (2007). CGHcall: calling aberrations for array CGH tumor profiles. *Bioinformatics*.

[12] Christensen B. C., Houseman E. A., Poage G. M. (2010). Integrated profiling reveals a global correlation between epigenetic and genetic alterations in mesothelioma. *Cancer Research*.

[13] Kilday J.-P., Mitra B., Domerg C. (2012). Copy number gain of 1q25 predicts poor progression-free survival for pediatric intracranial ependymomas and enables patient risk stratification: a prospective European clinical trial cohort analysis on behalf of the Children’s Cancer Leukaemia Group (CCLG). *Clinical Cancer Research*.

[14] Barrett T., Wilhite S. E., Ledoux P. (2013). NCBI GEO: Archive for functional genomics data sets—update. *Nucleic Acids Research*.

[15] R Core Team R: A language and environment for statistical computing. http://www.r-project.org/.

[16] Reimers M., Carey V. J. (2006). Bioconductor: an open source framework for bioinformatics and computational biology. *Methods in Enzymology*.

[17] Carvalho B. S., Irizarry R. A. (2010). A framework for oligonucleotide microarray preprocessing. *Bioinformatics*.

[18] Bauer S., Gagneur J., Robinson P. N. (2010). Going Bayesian: model-based gene set analysis of genome-scale data. *Nucleic Acids Research*.

[19] Ashburner M., Ball C. A., Blake J. A. (2000). Gene ontology: tool for the unification of biology. *Nature Genetics*.

[20] Kanehisa M., Goto S., Sato Y., Kawashima M., Furumichi M., Tanabe M. (2014). Data, information, knowledge and principle: back to metabolism in KEGG. *Nucleic Acids Research*.

[21] Cline M. S., Smoot M., Cerami E. (2007). Integration of biological networks and gene expression data using Cytoscape. *Nature protocols*.

[22] Wu G., Feng X., Stein L. (2010). A human functional protein interaction network and its application to cancer data analysis. *Genome Biology*.

[23] Nannya Y., Sanada M., Nakazaki K. (2005). A robust algorithm for copy number detection using high-density oligonucleotide single nucleotide polymorphism genotyping arrays. *Cancer Research*.

[24] Yin S., Xu L., Bonfil R. D. (2013). Tumor-initiating cells and FZD8 play a major role in drug resistance in triple-negative breast cancer. *Molecular Cancer Therapeutics*.

[25] Lu J.-P., Zhang J., Kim K. (2010). Human homolog of *Drosophila* Hairy and enhancer of split 1, Hes1, negatively regulates *δ*-catenin (*CTNND2*) expression in cooperation with E2F1 in prostate cancer. *Molecular Cancer*.

[26] Matthaios D., Zarogoulidis P., Balgouranidou I., Chatzaki E., Kakolyris S. (2011). Molecular pathogenesis of pancreatic cancer and clinical perspectives. *Oncology*.

[27] Kremmidiotis G., Baker E., Crawford J., Eyre H. J., Nahmias J., Callen D. F. (1998). Localization of human cadherin genes to chromosome regions exhibiting cancer-related loss of heterozygosity. *Genomics*.

[28] Jeanes A., Gottardi C. J., Yap A. S. (2008). Cadherins and cancer: how does cadherin dysfunction promote tumor progression?. *Oncogene*.

[29] Cavallaro U., Christofori G. (2004). Cell adhesion and signalling by cadherins and Ig-CAMs in cancer. *Nature Reviews Cancer*.

[30] Pelin K., Hirvonen A., Linnainmaa K. (1994). Expression of cell adhesion molecules and connexins in gap junctional intercellular communication deficient human mesothelioma tumour cell lines and communication competent primary mesothelial cells. *Carcinogenesis*.

[31] Ivanov S. V., Miller J., Lucito R. (2009). Genomic events associated with progression of pleural malignant mesothelioma. *International Journal of Cancer*.

[32] Sekido Y. (2013). Molecular pathogenesis of malignant mesothelioma. *Carcinogenesis*.

[33] Lin Z., Yin P., Reierstad S. (2010). Adenosine A1 receptor, a target and regulator of estrogen receptoralpha action, mediates the proliferative effects of estradiol in breast cancer. *Oncogene*.

[34] Paul S., Elsinga P. H., Ishiwata K., Dierckx R. A. J. O., Van Waarde A. (2011). Adenosine A1 receptors in the central nervous system: their functions in health and disease, and possible elucidation by PET imaging. *Current Medicinal Chemistry*.

[35] Brocke K. S., Staufner C., Luksch H. (2010). Glutamate receptors in pediatric tumors of the central nervous system. *Cancer Biology and Therapy*.

[36] Buckingham S. C., Campbell S. L., Haas B. R. (2011). Glutamate release by primary brain tumors induces epileptic activity. *Nature Medicine*.

[37] Bridges R. J., Natale N. R., Patel S. A. (2012). System x_c_
^−^ cystine/glutamate antiporter: an update on molecular pharmacology and roles within the CNS. *British Journal of Pharmacology*.

[38] Elsharkawy A. E., Abuamona R., Bergmann M., Salem S., Gafumbegete E., Röttger E. (2013). Cortical anaplastic ependymoma with significant desmoplasia: a case report and literature review. *Case Reports in Oncological Medicine*.

[39] Ménard C., Valastro B., Martel M.-A. (2005). AMPA receptor phosphorylation is selectively regulated by constitutive phospholipase A_2_ and 5-lipoxygenase activities. *Hippocampus*.

[40] Figarella-Branger D., Lepidi H., Poncet C. (1995). Differential expression of cell adhesion molecules (CAM), neural CAM and epithelial cadherin in ependymomas and choroid plexus tumors. *Acta Neuropathologica*.

[41] Zhou J., Li J., Chen J., Liu Y., Gao W., Ding Y. (2009). Over-expression of CDH22 is associated with tumor progression in colorectal cancer. *Tumor Biology*.

[42] Johnson R. A., Wright K. D., Poppleton H. (2010). Cross-species genomics matches driver mutations and cell compartments to model ependymoma. *Nature*.

[43] de Bont J. M., Packer R. J., Michiels E. M., den Boer M. L., Pieters R. (2008). Biological background of pediatric medulloblastoma and ependymoma: a review from a translational research perspective. *Neuro-Oncology*.

